# Transient Receptor Potential Vanilloid 1 Expression Mediates Capsaicin-Induced Cell Death

**DOI:** 10.3389/fphys.2018.00682

**Published:** 2018-06-05

**Authors:** Ricardo Ramírez-Barrantes, Claudio Córdova, Sebastian Gatica, Belén Rodriguez, Carlo Lozano, Ivanny Marchant, Cesar Echeverria, Felipe Simon, Pablo Olivero

**Affiliations:** ^1^Laboratorio de Estructura y Función Celular, Escuela de Medicina, Facultad de Medicina, Universidad de Valparaíso, Valparaíso, Chile; ^2^Departamento de Ciencias Biologicas, Facultad de Ciencias de la Vida, Universidad Andres Bello, Santiago, Chile; ^3^Millennium Institute on Immunology and Immunotherapy, Santiago, Chile; ^4^Centro Integrativo de Biología y Química Aplicada, Universidad Bernardo O’Higgins, Santiago, Chile

**Keywords:** TRPV1, capsaicin, cell death, mitochondria, necrosis, calcium

## Abstract

The transient receptor potential (TRP) ion channel family consists of a broad variety of non-selective cation channels that integrate environmental physicochemical signals for dynamic homeostatic control. Involved in a variety of cellular physiological processes, TRP channels are fundamental to the control of the cell life cycle. TRP channels from the vanilloid (TRPV) family have been directly implicated in cell death. TRPV1 is activated by pain-inducing stimuli, including inflammatory endovanilloids and pungent exovanilloids, such as capsaicin (CAP). TRPV1 activation by high doses of CAP (>10 μM) leads to necrosis, but also exhibits apoptotic characteristics. However, CAP dose–response studies are lacking in order to determine whether CAP-induced cell death occurs preferentially via necrosis or apoptosis. In addition, it is not known whether cytosolic Ca^2+^ and mitochondrial dysfunction participates in CAP-induced TRPV1-mediated cell death. By using TRPV1-transfected HeLa cells, we investigated the underlying mechanisms involved in CAP-induced TRPV1-mediated cell death, the dependence of CAP dose, and the participation of mitochondrial dysfunction and cytosolic Ca^2+^ increase. Together, our results contribute to elucidate the pathophysiological steps that follow after TRPV1 stimulation with CAP. Low concentrations of CAP (1 μM) induce cell death by a mechanism involving a TRPV1-mediated rapid and transient intracellular Ca^2+^ increase that stimulates plasma membrane depolarization, thereby compromising plasma membrane integrity and ultimately leading to cell death. Meanwhile, higher doses of CAP induce cell death via a TRPV1-independent mechanism, involving a slow and persistent intracellular Ca^2+^ increase that induces mitochondrial dysfunction, plasma membrane depolarization, plasma membrane loss of integrity, and ultimately, cell death.

## Introduction

Transient receptor potential (TRP) channels belong to a polymodal family of ion channels that act as molecular transducers and integrators of a variety of environmental physicochemical stimuli, such as temperature, osmotic pressure, mechanical stress, and exogenous and endogenous ligands (Ramsey et al., 2006; Latorre et al., 2007, 2009). TRP channels play an essential role in multiple physiological and pathological cellular processes, such as proliferation, differentiation, and death progression (Shimizu et al., 2004; Shirakawa et al., 2008; Carrasco et al., 2018). Deregulated activation of TRP channels from the vanilloid (TRPV) family has been directly implicated in cell death (Macho et al., 1999; Amantini et al., 2009; Chen et al., 2012). TRPV1 has been detected in a variety of organs, such as the brain, testes, lungs, and heart. (Hayes et al., 2000; Randhawa and Jaggi, 2018). TRPV1 is widely expressed in dorsal root ganglion (DRG) and trigeminal neurons.

TRPV1 is activated by pain-inducing stimuli, including inflammatory endovanilloids, TNF-α, TGF-β, heat (37–42°C), acids (pH < 6.3), and pungent exovanilloids, such as capsaicin (CAP) or resiniferatoxin (Caterina et al., 1997; Tominaga et al., 1998; Jordt et al., 2000; Olah et al., 2001, 2002; Latorre et al., 2007; Ma et al., 2011; Utreras et al., 2013; Rozas et al., 2016). TRPV1 activation by CAP is antagonized by the synthetic organic compound capsazepine (CPZ). At the cellular level, TRPV1 activation by high doses of CAP leads not only to necrotic processes with membrane bleb formation (Pecze et al., 2013; Wu et al., 2014) but also to apoptosis through caspase-3 activation and mitochondrial membrane potential attenuation (Ziglioli et al., 2009; Sun et al., 2014; Çiğ and Nazıroğlu, 2015). However, CAP dose–response studies are lacking in order to understand whether CAP-induced cell death occurs preferentially via necrosis or apoptosis. Thus, the relationship between CAP concentration and CAP-induced TRPV1-mediated cell death is not completely understood.

Increased TRPV1 activity induces high levels of cytosolic Ca^2+^, generating mitochondrial membrane depolarization and decreased cell viability (Thomas et al., 2007). Furthermore, TRPV1 activation triggers apoptotic cell death in neuron-rich cultures from rat cerebral cortex via Ca^2+^ channels opening, allowing Ca^2+^ influx (Shirakawa et al., 2008). However, it is not known whether cytosolic Ca^2+^ and mitochondrial dysfunction participate in CAP-induced TRPV1-mediated cell death. Thus, we focused on investigating the underlying mechanisms involved in CAP-induced TRPV1-mediated cell death, the dependence of CAP dose, and the participation of mitochondrial dysfunction and cytosolic Ca^2+^ increase.

Using an analytical three-state model (O’Neill et al., 2011) to describe the mechanistic sequential progression from a state of health to a state of death, we found that TRPV1 stimulation with 10 μM CAP significantly induces necrosis-like cell death characterized by extensive cell membrane damage but without affecting mitochondrial function. Interestingly, 100 μM CAP induced a different pattern for cell death, characterized by mitochondrial dysfunction and is independent of TRPV1 activity, resembling an apoptosis-like death pattern. Furthermore, we found that TRPV1 stimulation with 1 μM CAP induces a TRPV1-dependent fast and transient intracellular Ca^2+^ increase, while 10 μM CAP induces a fast and persistent increase, which can be explained by the combination of two intracellular Ca^2+^ signals, a TRPV1-dependent fast and transient increase that is inhibited by CPZ, and a slow, persistent, and TRPV1-independent rise of intracellular Ca^2+^. Finally, we demonstrated that 10 μM CAP induces plasma membrane depolarization via an influx of Ca^2+^ and Na^+^ from the extracellular space.

Our results show further mechanistical insights detailing how CAP induces TRPV1-dependent and independent cell death. Low concentrations of CAP (1 μM) induce a fast and transient increase in intracellular Ca^2+^, which leads to plasma membrane depolarization, thereby compromising plasma membrane integrity, and ultimately driving cell physiology to a state of death but without mitochondrial dysfunction. Meanwhile, 10 and 100 μM CAP induce a slow but persistent increase in intracellular Ca^2+^, which leads not only to plasma membrane depolarization but also to mitochondrial dysfunction, and ultimately cell death. Thus, CAP is shown to activate two independent pathways of Ca^2+^ homeostasis leading to cell death by necrosis or apoptosis.

## Materials and Methods

### Cell Culture

HeLa cells were obtained from ATCC (Manassas, VA, United States). The culture medium used was Dulbecco’s Modified Eagle Medium/F12 supplemented with 10% fetal bovine serum and 50 U/ml penicillin–streptomycin. Cells were incubated in a conventional incubator at 37°C and a 95% air/5% CO_2_ atmosphere.

### HeLa Cells Stably Transfected With TRPV1 (st-TRPV1 HeLa Cells)

HeLa cells were cultured at 70–80% confluence and then were transfected with pcDNA3.1 containing the full length of rat TRPV1 (GenBankTM accession no. NM031982) using Lipofectamine (ThermoFisher). Transfected cells were selected using Geneticin (Sigma-Aldrich, St. Louis, MO, United States, 800 mg/mL) to generate a stable cell line encoding TRPV1. Stable TRPV1 expression was checked by RT-PCR and flow cytometry weekly (Supplementary Figure S1).

### Analysis of Quantitative Cell Death by Flow Cytometry

HeLa cells were exposed to different experimental conditions in Dulbecco’s Modified Eagle Medium/F12 supplemented with 1% bovine serum albumin instead of fetal bovine serum. Cellular death was determined as described in the literature (Darzynkiewicz et al., 1982) and analyzed according to a three-state model of cell death (O’Neill et al., 2011). Briefly, cell cultures were stained with 10 μM rhodamine 123 (Rho123, Invitrogen, Carlsbad, CA, United States) to assess mitochondrial membrane potential, and propidium iodide (PI, 10 μg/ml (Sigma-Aldrich, St. Louis, MO, United States) to assess plasma membrane integrity. Both measures were analyzed by flow cytometry (FACScalibur, BD Biosciences, CA, United States). A minimum of 10,000 cells/sample were analyzed to evaluate mitochondrial function and membrane permeability. Fluorescence intensity analysis was performed using FlowJo software (Tree Star, Inc., Ashland, OR, United States). Cell state was operationally defined with the following first-order rate process: Alive (*A*) ↔ Vulnerable (*V*) ↔ Dead (*D*). To determine probability for the *A* state (P*_A_*), data were normalized with respect to internal controls using the following equation: *P_A_* = *X* – *C_D_*/*C_A_* -*C_D_*, where *X* corresponds to living cells in each experimental condition, *C_D_* corresponds to living cells in the presence of 10% ethanol to induce cell death, and *C_A_* corresponds to living cells without treatment.

### Ca^2+^ Imaging

Cell cultures were loaded with Fura-2 AM (Molecular Probes, Eugene, OR, United States) for 30 min at room temperature in buffer solution [130 mM NaCl, 5.4 mM KCl, 2.5 mM CaCl_2_, 0.8 mM MgCl_2_, 5.6 mM glucose, 10 mM HEPES, pH 7.4 (adjusted with Tris base)], rinsed, and allowed to equilibrate for 5–10 min. Next, HeLa cells were cultured on 12-mm glass cover slips in a recording chamber mounted on an epifluorescence Olympus IX81 microscope (Olympus, Japan) equipped with a multiple-excitation filter wheel. CAP-induced activity was recorded for a minimum recording time of 2 s. Maximum resolution was obtained with a Plan Apo 40X 1.3 NA oil objective lens.

### Membrane Potential Measurement

Cell cultures were equilibrated using DiBAC_4_(3) [Molecular Probes, Eugene, OR, United States] as described previously (Kunz et al., 2006). This anionic fluorescent dye is distributed across the plasma membrane relative to the membrane potential following Nernst’s equation (Olivero et al., 2008). DiBAC_4_(3) (200 nM) was applied extracellularly for approximately 20 min to ensure dye distribution across the cell membrane. Changes in fluorescence intensity were monitored by sampling every 10 s for 30 min with a 515 nm excitation filter and an emission filter of at least 600 nm. Fluorescence data were transformed to mV using a calibration curve from HeLa cells as described previously (Krasznai et al., 1995).

### RT-PCR

Total RNA from parental HeLa cells and cells transfected with TRPV1 was extracted with TRIzol (Invitrogen, Carlsbad, CA, United States), and reverse transcription was performed to create a cDNA library using reverse transcriptase M-MLV (Invitrogen, Carlsbad, CA, United States). An equal amount of RNA was used as template in each reaction. The PCR reactions were performed using GoTaq Master Mix (Promega Corp., Madison, WI, United States) following the manufacturer’s instructions.

### Immunodetection by Flow Cytometry

HeLa cells were collected by trypsinization and fixed with 4% paraformaldehyde for 30 min. Next, the cells were blocked and permeabilized using a PBS solution with 5% bovine serum albumin (Merck KGaA, Darmstadt, Germany) and 2% Tween-20 (Merck KGaA, Darmstadt, Germany) for 1 h and then incubated with an anti-TRPV1 antibody (Santa Cruz Biotechnology, Inc., United States, 1:200) in blocking solution overnight at 4°C. After washing with PBS, the cells were incubated with anti-goat biotinylated secondary antibody (Jackson ImmunoResearch, United States, 1:500) for 1 h at 37°C. The cells were washed with PBS and incubated in the dark with streptavidin-Alexa Fluor 488 (Jackson ImmunoResearch, United States, 1:200) for 1 h at 37°C. The Alexa Fluor signal was measured with a 530/30 bandpass filter using an argon laser at 488 nm integrated into a FACScalibur flow cytometer (BD, Biosciences, CA, United States). Debris and duplets were excluded from the analysis, and a minimum of 10,000 cells were acquired in each experiment. Data were analyzed with FlowJo software (Tree Star, Inc., Ashland, OR, United States).

### Reagents

Cyanide-4-(trifluoromethoxy)phenylhydrazone, FCCP, ionomycin, rodamine123, and PI were obtained from Sigma-Aldrich (St. Louis, MO, United States). CAP and CPZ were obtained from Tocris Bioscience (Bristol, United Kingdom). Buffers, ethanol, and salts were purchased from Merck (Darmstadt, Germany).

### Data Analysis

All results are presented as the mean ± SD from at least three independent assays for each experimental condition. Fisher’s least significant difference test and an ANOVA test followed by the Bonferroni *post hoc* test were used to compare multiple groups using Statgraphics Plus 5.0 (GraphPad Software, Inc., San Diego, CA, United States). A *p*-value < 0.05 was used to indicate statistical significance.

## Results

### TRPV1 Expression Increases CAP-Induced Cell Death

**FIGURE 1:**
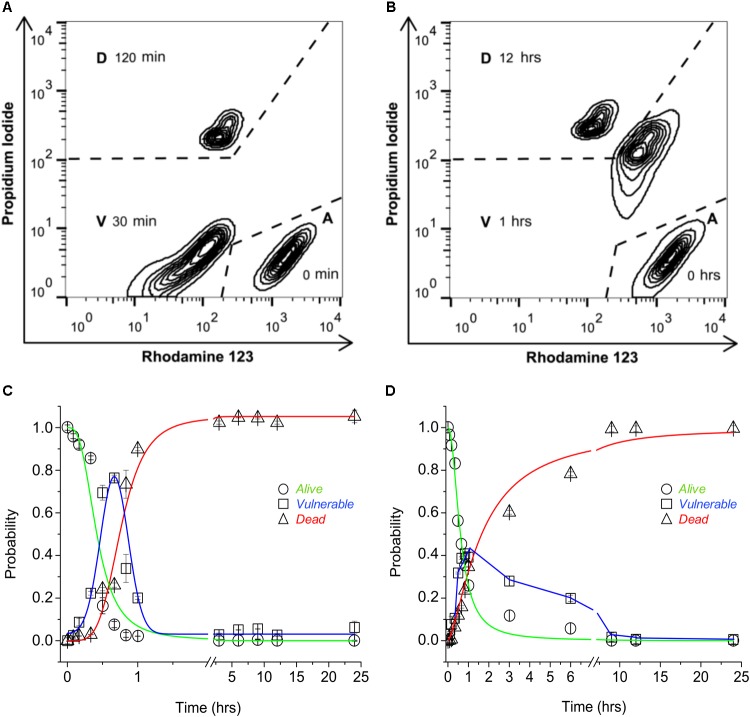
Three-state model of cell death. Representative flow cytometry dot plot output depicting 24-h exposure of non-transfected wt-HeLa cell phenotype exposed to 10 μM FCCP **(A)** and 10% ethanol **(B)**. Cell death progression was evaluated using PI (to assess plasma membrane integrity) and Rho123 (to assess mitochondrial membrane potential). Cell state progression is symbolized as *A* (alive), *V* (vulnerable), and *D* (dead). Replicate experiments [from **(A,B)**] were performed for different times between 0 and 24 h, and were normalized against *P_A_* = 1 to determine the probability for cell state [**(C,D)** respectively]. Data are shown as mean ± SEM (*n* = 9).

The participation of TRPV1 expression and activity to sensitize cells to CAP-induced death was tested using a three-state cell death model. The three-state model [alive (*A*), vulnerable (*V*), and dead (*D*)] was established by means of flow cytometry dot plot analysis to determine cell death with or without mitochondrial involvement. Wild-type HeLa (wt-HeLa) cells were exposed to 10 μM FCCP (**Figure 1A**) for 0, 30, and 120 min, and 10% ethanol (**Figure 1B**) for 0, 1, and 12 h. The three-state cell death model displayed a pronounced progression from *A* (PI^low^Rho123^hi^), to *V* (PI^low^Rho123^low^, or PI^hi^Rho123^hi^), to *D (*PI^hi^Rho123^low^; **Figures 1A,B**). The induction of an intermediate PI^low^Rho123^low^ *V*-phenotype indicates loss of mitochondrial membrane potential without plasma membrane disruption (**Figure 1A**), while PI^hi^Rho123^hi^ *V*-phenotype indicates plasma membrane disruption, but without loss of mitochondrial membrane potential (**Figure 1B**). Cell phenotype did not remain constant, and the proportion of cells in the three states varied over time. Cells achieved a full phenotype shift toward the *D* state after 3 h of exposure to FCCP (**Figure 1C**) and after 9 h of exposure to 10% ethanol (**Figure 1D**). Cells reached the intermediate *V* state at approximately 1 h of exposure to either FCCP or 10% ethanol, and P*_A_* was greater for cells exposed to FCCP than to 10% ethanol. Therefore, HeLa cell physiology appeared more sensitive to mitochondrial dysfunction than to plasma membrane disruption, as shown with P*_A_* progression curves.

**FIGURE 2:**
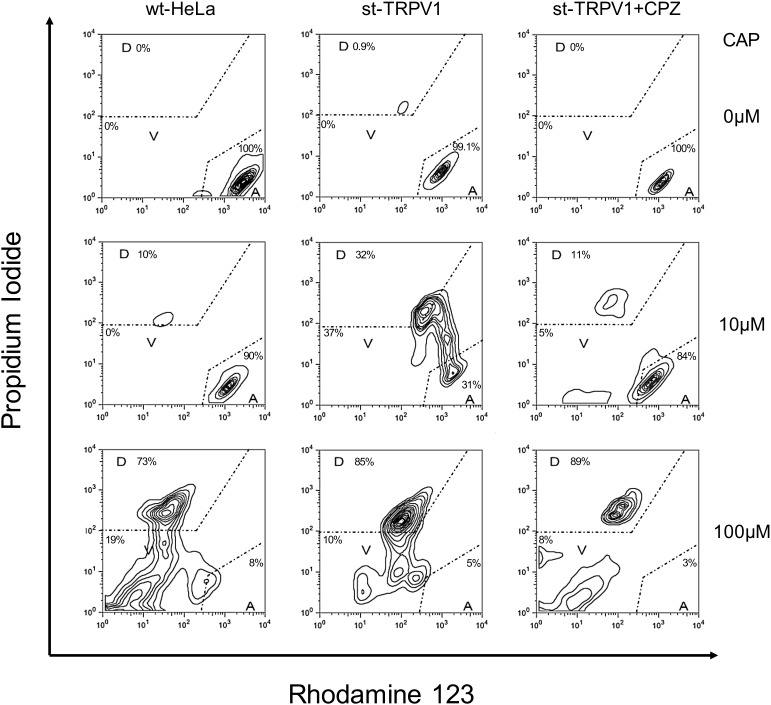
TRPV1 expression increases CAP-induced cell death. Representative flow cytometry dot plot output and quantification depicting wt-HeLa cells and st-TRPV1 HeLa cells exposed to 0, 10, and 100 μM CAP for 24 h in the presence or absence of 10 μM CPZ. Cell death progression was evaluated using PI (to assess plasma membrane integrity) and Rho123 (to assess mitochondrial membrane potential). Cell state progression is symbolized as *A* (alive), *V* (vulnerable), and *D* (dead). Data are shown as mean ± SEM (*n* = 6).

Once the three-state cell death model was established, we investigated the effect of TRPV1 expression in HeLa cells stably transfected with TRPV1 (st-TRPV1). Transfection efficiency in generating the st-TRPV1 was confirmed at the mRNA level by RT-PCR and at the protein level by flow cytometry (Supplementary Figure S1). Flow cytometry analysis revealed that in the absence of CAP, wt-HeLa cells predominantly exhibited a phenotype consistent with the *A* state (PI^low^Rho123^hi^), without plasma membrane disruption or mitochondrial dysfunction (**Figure 2**, upper-left panel). Similar results were observed for st-TRPV1 HeLa cells in the absence (**Figure 2**, upper-middle panel) or presence of CPZ (**Figure 2**, upper-right panel), and in wt-HeLa cells exposed to 10 μM CAP (**Figure 2**, middle-left panel). However, st-TRPV1 HeLa cells exposed to 10 μM CAP showed a phenotype (PI^low-hi^Rho123^hi^) predominantly indicative of plasma membrane loss of integrity without mitochondrial dysfunction (**Figure 2**, middle-middle panel), a phenotype that resembles a necrosis-like cell death. Interestingly, st-TRPV1 HeLa cells pre-treated with 10 μM CPZ and then treated with 10 μM CAP exhibited a cell phenotype consistent with the *A* state (**Figure 2**, middle-right panel), suggesting that 10 μM CPZ is able to prevent cell death. Wt-HeLa cells exposed to 100 μM CAP showed a phenotype (PI^hi^Rho123^low^) predominantly indicative of plasma membrane loss of integrity and severe mitochondrial dysfunction (**Figure 2**, lower-left panel). Similarly, st-TRPV1 HeLa cells exposed to 100 μM CAP exhibited a phenotype (PI^hi^Rho123^mid^) consistent with the *D* state with both plasma membrane disruption and mitochondrial failure (**Figure 2**, lower-middle panel), indicative of an apoptosis-like cell death. Notably, pre-treatment with 10 μM CPZ of st-TRPV1 HeLa cells exposed to 100 μM CAP was not effective protecting the cells from CAP challenge, showing a phenotype (PI^hi^Rho123^mid^) consistent with the *D* state (**Figure 2**, lower-right panel). Thus, these results indicate that 10 μM CAP induces TRPV1-mediated cell death without affecting mitochondrial physiology, while 100 μM CAP induces cell death in a TRPV1-independent fashion, characterized by mitochondrial potential dysfunction and plasma membrane disruption. Phase-contrast images highlighting the main morphological features of each condition for wt-HeLa and st-TRPV1 HeLa cells were documented and summarized in Supplementary Figure S2.

**FIGURE 3:**
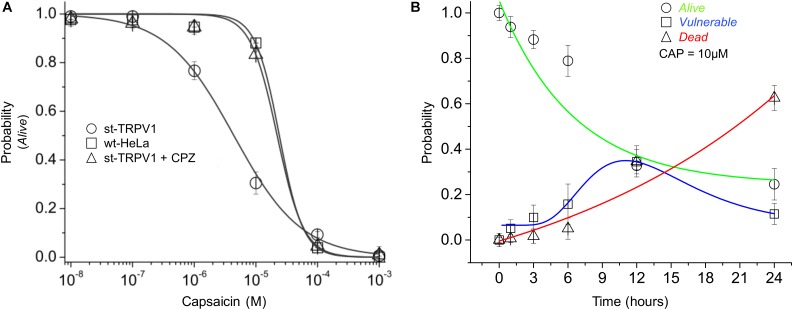
Dose- and time-response of CAP-induced cell death. **(A)** Probability of cell state *A* (*P_A_*) of wt-HeLa cells and st-TRPV1 HeLa cells in the presence or absence of 10 μM CPZ exposed to several concentration of CAP (1⋅10^-3^ to 1⋅10^-8^ M) for 24 h. **(B)** Probability of cell state progression of st-TRPV1 HeLa cells exposed to 10 μM CAP for 0, 1, 3, 6, 12, and 24 h. Data are shown as mean ± SEM (*n* = 5). ^∗^*p* < 0.01.

To investigate the dose–response effect of CAP, st-TRPV1 HeLa cells were exposed to increasing doses of CAP for 24 h. The results showed that CAP has a sensitizing effect over st-TRPV1 HeLa cells, decreasing the P*_A_* when compared to wt-HeLa cells (P_A50_ from ∼3.5⋅10^-5^ to ∼4.5⋅10^-6^ μM CAP). Interestingly, addition of the TRPV1 competitive antagonist CPZ (which blocks CAP-induced Ca^2+^ uptake through TRPV1) to CAP treated st-TRPV1 HeLa cells completely overturned the original sensitizing effect of CAP, contributing to cell resistance to a level close to wt-HeLa cells response (**Figure 3A**). As a next step, we studied the time–response (0–24 h) of st-TRPV1 cells exposed to 10 μM CAP. The proportion of st-TRPV1 cells in the *V* state was maximal after 12 h of exposure to 10 μM CAP. The *A* state in st-TRPV1 cells reached 50% after ∼7 h of CAP exposure, while the *D* state increased steadily (**Figure 3B**). These results indicate that the CAP-induced cell-state transition is mediated by TRPV1.

### TRPV1 Expression Increases Intracellular Calcium Level in Response to CAP

**FIGURE 4:**
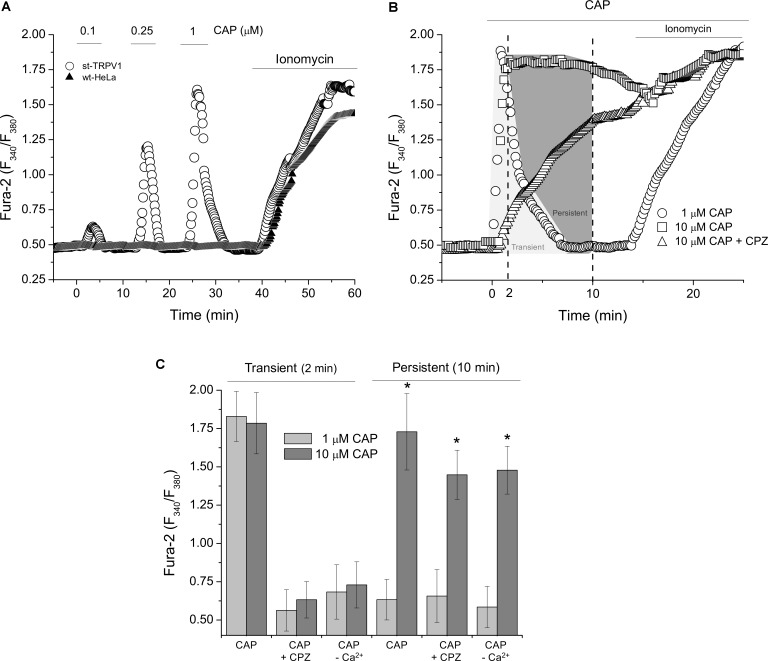
TRPV1 expression increases intracellular calcium level in response to CAP. **(A)** Dose–response curve of ratiometric assay recordings for [Ca^2+^]_I_ in wt-HeLa cells and st-TRPV1 HeLa cells exposed to 0.1, 0.25, and 1 μM CAP for 60 min. Representative experiments from five independent experiments. **(B)** Dose–response curve of ratiometric assay recordings for [Ca^2+^]_I_ in st-TRPV1 HeLa cells exposed to 1 and 10 μM CAP in the presence or absence of 10 μM CPZ for 20 min. Representative experiments from five independent experiments. 10 μM ionomycin was added at the end of experiments **(A)** and **(B)** as positive control. **(C)** Cross-sectional ratiometric [Ca^2+^]_I_ in st-TRPV1 HeLa cells exposed to 1 and 10 μM CAP at times 2 and 10 min after CAP exposition, in the presence or absence of 10 μM CPZ or Ca^2+^-free condition. Data are shown as mean ± SEM (*n* = 15). ^∗^*p* < 0.01.

To investigate the intracellular effect of CAP-mediated TRPV1 stimulation, we measured Ca^2+^ dynamics with ratiometric assays. Measurements of intracellular Ca^2+^ levels showed that wt-HeLa cells were irresponsive to CAP stimulation. However, st-TRPV1 HeLa cells were able to respond to CAP treatment exhibiting transient and dose-dependent increases in intracellular calcium concentration ([Ca^2+^]_I_; **Figure 4A**). Addition of 1 μM CAP to st-TRPV1 HeLa cells showed a fast and transient rise in [Ca^2+^]_I_ after 2 min of exposure to CAP reaching basal levels shortly before 10 min (**Figure 4B**). However, st-TRPV1 HeLa cells treated with 10 μM CAP showed a fast and persistent increase in [Ca^2+^]_I_, without return to basal levels after 10 min. Interestingly, when the latter cells were simultaneously exposed to 10 μM CPZ, the fast increase in [Ca^2+^]_I_ was prevented, showing a slow and constant increase in [Ca^2+^]_I_ reaching levels similar to those observed in the absence of CPZ (**Figure 4B**). Thus, 10 μM CAP elicits two Ca^2+^ signals combined, a TRPV1-dependent fast and transient increase and another slow and persistent Ca^2+^ increase, without participation of TRPV1. As a positive control, cells were responsive to ionomycin (**Figures 4A,B**).

Whether the increase in intracellular Ca^2+^ levels was mediated by TRPV1, we were prompted to determine the participation of extracellular Ca^2+^ on the CAP-induced increase on intracellular Ca^2+^ levels. **Figure 4C** depicts both the transient and the persistent increases in [Ca^2+^]_I_ from experiments as shown in **Figure 4B**. St-TRPV1 HeLa cells exposed to 1 μM CAP showed a transient, but not persistent increase in [Ca^2+^]_I_, while cells exposed to 10 μM CAP showed a persistent increase in [Ca^2+^]_I_. The addition of 10 μM CPZ prevented both the transient increase in [Ca^2+^]_I_ independent of CAP concentration, but CPZ failed into inhibit the persistent increase in [Ca^2+^]_I_ when 10 μM CAP was present. Interestingly, after removal of extracellular Ca^2+^, transient Ca^2+^ increases were prevented for both 1 and 10 μM CAP treatments, suggesting that CAP-induced transient increases in [Ca^2+^]_I_ are dependent on extracellular Ca^2+^ and considering that it is CPZ-sensitive, this influx is mediated TRPV1. However, after extracellular Ca^2+^ depletion, 10 μM CAP was still able to stimulate an increase in [Ca^2+^]_I_, potentially due to a secondary mechanism involving Ca^2+^ mobilization from an intracellular storage compartment (**Figure 4C**).

### TRPV1 Expression Induces Plasma Membrane Depolarization in Response to CAP

**FIGURE 5:**
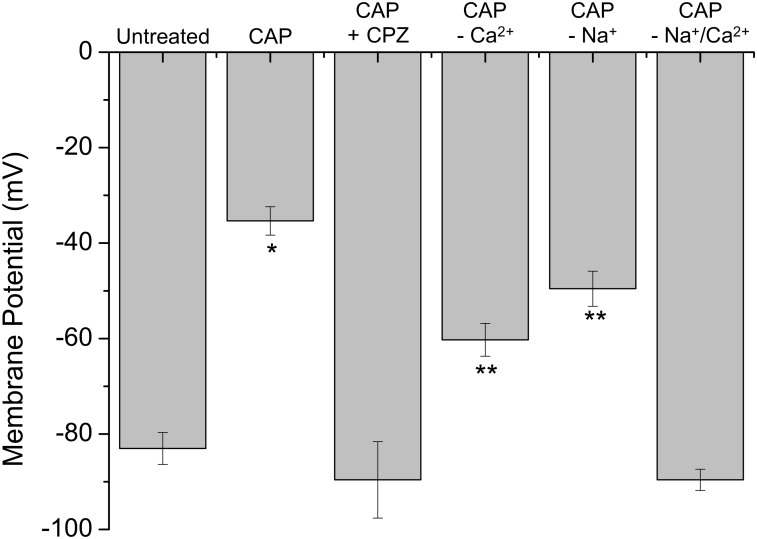
TRPV1 expression induces plasma membrane depolarization in response to CAP. Membrane potential measured by the change in DiBAC_4_(3) fluorescence intensity in st-TRPV1 HeLa cells exposed to 10 μM CAP for 30 min, in the presence or absence of 10 μM CPZ, Ca^2+^-free condition, or Na^+^-free condition. Data are shown as mean ± SEM (*n* = 5). ^∗^*p* < 0.01 and ^∗∗^*p* < 0.05.

Considering that stimulation of st-TRPV1 with CAP generated a fast increase in [Ca^2+^]_I_ (**Figure 4B**) and that CAP induced a disruption in membrane integrity but not mitochondrial function (**Figure 2**, middle panel), we determined whether CAP challenge could depolarize plasma membrane potential. St-TRPV1 HeLa cells exposed to 10 μM CAP for 30 min increased the fluorescence of the membrane potential fluorescent indicator DIBAC_4_(3), suggesting that exposure to 10 μM CAP induced a significant depolarization of the plasma membrane from approximately –80 mV in non-treated st-TRPV1 HeLa cells to –35 mV (**Figure 5**). Interestingly, selective inactivation of TRPV1 with 10 μM CPZ prevented the CAP-induced plasma membrane depolarization, suggesting that TRPV1 participates in plasma membrane depolarization induced by CAP.

To investigate whether CAP-induced plasma membrane depolarization requires external Ca^2+^, st-TRPV1 HeLa cells were exposed to 10 μM CAP in the absence of extracellular Ca^2+^. These results showed that in the absence of external Ca^2+^, CAP-induced plasma membrane depolarization was significantly decreased. Often, intracellular Ca^2+^ increases are followed by increases in intracellular Na^+^, which severely contribute to plasma membrane depolarization (**Figure 5**; Castro et al., 2006). To test this possibility, st-TRPV1 HeLa cells were exposed to 10 μM CAP in the presence of a culture medium depleted of Na^+^ by means of replacing Na^+^ with the non-permeable cation NMDG^+^, thereby maintaining osmolarity and tonicity constant. These results showed that in the absence of external Na^+^, the CAP-induced plasma membrane depolarization was significantly decreased. Remarkably, CAP-induced plasma membrane depolarization was completely prevented when both Ca^2+^ and Na^+^ were absent from the extracellular medium (**Figure 5**). Thus, CAP-induced plasma membrane depolarization appears to be an additive effect of Ca^2+^ and Na^+^ influxes. DIBAC_4_(3) efficiency to measure plasma membrane potential was validated using valinomycin and gramicidin (Supplementary Figure S3).

## Discussion

This study highlights how TRPV1 activity is required to induce cell death. Here, we suggest that TRPV1 stimulation with CAP induces necrotic-like cell death without mitochondrial dysfunction, in a mechanism that involves a fast and transient increase in intracellular Ca^2+^, leading to plasma membrane depolarization and a loss of plasma membrane integrity. Interestingly, 100 μM CAP generates mitochondrial dysfunction and TRPV1-independent apoptotic-like cell death.

TRPV1 expression exhibits dose-dependent cytotoxic effects, including mitochondrial store-dependent Ca^2+^ overload (Lam et al., 2007; Hu et al., 2008; Davies et al., 2010), membrane bleb formation (Pecze et al., 2013; Wu et al., 2014), pyknotic nuclear fragmentation, cytochrome *c* release from mitochondria, caspase-3 activation (Davies et al., 2010), and cell viability (Maeno et al., 2000; Bortner and Cidlowski, 2002; Stutzin and Hoffmann, 2006; Lam et al., 2007; Panayiotidis et al., 2010). Indeed, TRPV1 activation promises therapeutic use by rapidly and selectively inducing necrosis in TRPV1-expressing nociceptive neurons (Olah et al., 2001), thereby inducing analgesia most likely via Ca^2+^-mediated cytotoxicity (Marsch et al., 2007; Gunthorpe and Chizh, 2009; Lambert, 2009). Furthermore, the analgesic effects of CAP – via TRPV1 activation – are associated with the inhibition of hyperpolarization-activated cation currents (*I*_h_), which depend on intracellular Ca^2+^ mobilization (Kwak, 2012). Optimal mitochondrial physiology maintains a low cytoplasmic Ca^2+^ concentration through mitochondrial refilling and/or ATP-dependent Ca^2+^ compartmentalization processes (Varikmaa et al., 2013). Thus, CAP can induce sustained Ca^2+^ increases, likely via the release of Ca^2+^ stores caused by mitochondrial failure or mitochondrial fission.

Stable TRPV1 expression and stimulation induces membrane depolarization through an increase in intracellular Ca^2+^. Although transient increases in Ca^2+^ did not induce a change in cell state, heavy stimulation of TRPV1 with 100 μM CAP was able to trigger a toxic Ca^2+^ overload, likely due to intracellular mitochondrial Ca^2+^ release. Mitochondrial Ca^2+^ exchange with the cytoplasm has been previously reported (Malli et al., 2003) and Ca^2+^ uptake has been proposed to generate microdomains of low Ca^2+^ concentrations across the cytoplasm (Youle and van der Bliek, 2012). In fact, mitochondrial function has been found to be finely regulated by Ca^2+^-dependent ion channels, capable of regulating the electrochemical gradients required to mobilize Ca^2+^ into intracellular stores (Malli et al., 2003). Thus, the loss of membrane potential is likely induced by the activation of TRPV1 and a massive Ca^2+^ influx, leading to CAP-induced cell death.

Endogenous TRPV1 expression in many tissues, such as the brain, heart, skin, and testis, has been reported to play a role in cell death (Kunert-Keil et al., 2006; Marsch et al., 2007). For example, TRPV1-mediated neurotoxicity has been reported in a large spectrum of assays involving primary cultures, as well as tissues, organs, and *in toto* experiments (Shin et al., 2003; Cernak et al., 2004; Shirakawa et al., 2008). In fact, direct application of CAP to the substantia nigra can induce the depletion of dopaminergic neurons (Kim et al., 2005). Moreover, deregulated TRPV1 activation by endogenous agonists induces the loss of hippocampal neurons and an impairment of cognitive functions (Cernak et al., 2004). These findings, along with observations by other authors, raise the possibility that TRPV1 receptors may be inducing cell death via oxidative stress (Macho et al., 1999; Movsesyan et al., 2004; Lam et al., 2007; Shirakawa et al., 2008), mitochondrial disruption (Macho et al., 1999; Shin et al., 2003), and intracellular Ca^2+^ overload (Chard et al., 1995; Cernak et al., 2004; Lam et al., 2007; Shirakawa et al., 2008). Nevertheless, this conjecture falls beyond the scope of this work and warrants further investigation in neuronal cells.

Taken together, the results shown in this study suggest that the expression and specific activation of TRPV1 can induce TRPV1-dependent Ca^2+^ signaling modifications that lead to a plasma membrane potential depolarization contributing to cell death.

## Author Contributions

RR-B, CC, SG, BR, CL, IM, CE, FS, and PO critically revised and edited this manuscript. RR-B, FS, and PO participated in the research design. RR-B, CC, BR, CL, IM, and PO conducted the experiments and performed data analyses. R-RB, SG, FS, and PO contributed to the figure design. RR-B, SG, IM, CE, FS, and PO wrote the paper.

## Conflict of Interest Statement

The authors declare that the research was conducted in the absence of any commercial or financial relationships that could be construed as a potential conflict of interest.
