# Supplementary material for: Transient Receptor Potential Vanilloid 1 Expression Mediates Capsaicin-Induced Cell Death
Source: Front Physiol. 2018 Jun 5;9:682. doi: 10.3389/fphys.2018.00682 (PMC5996173; doi:10.3389/fphys.2018.00682)
Supplement: Supplementary file 1 [file Data_Sheet_1.PDF]

frontiers

Supplementary Material

Transient Receptor Potential Vanilloid 1 Expression Mediates Capsaicin-Induced Cell Death

Ricardo Ramírez-Barrantes[1], Claudio Córdova[1], Sebastian Gatica[2,4], Belén Rodriguez[1], Carlo Lozano[1], Ivanny Marchant[1], Cesar Echeverria[3], Felipe Simon[2,4,*], Pablo Olivero[1,*]

[1]CIMT - EFCLab, Facultad de Medicina, Universidad de Valparaíso, Hontaneda 2664, 2341386, Valparaíso, Chile.
[2]Facultad de Ciencias de la Vida, Universidad Andres Bello, Ave. Republica 239, 8370146, Santiago, Chile.
[3]Centro Integrativo de Biología y Química Aplicada, Universidad Bernardo OHiggins, General Gana 1702, 8370854, Santiago, Chile.
[4]Millennium Institute on Immunology and Immunotherapy, Ave. Alameda 340, 8331150, Santiago, Chile.

Running title: TRPV1-mediates CAP-induced death

*: Corresponding Authors:

Felipe Simon, PhD
Facultad de Ciencias de la Vida
Universidad Andres Bello,
Ave. Republica 239, 8370134,
Santiago, Chile.
Phone: +562 661 5653,
e-mail: fsimon@unab.cl

Pablo Olivero, PhD
CIMT - EFCLab,
Facultad de Medicina,
Universidad de Valparaíso,
Hontaneda 2664, 2341386
Valparaíso, Chile
Phone: +5632 250 7322
e-mail: pablo.olivero@uv.cl

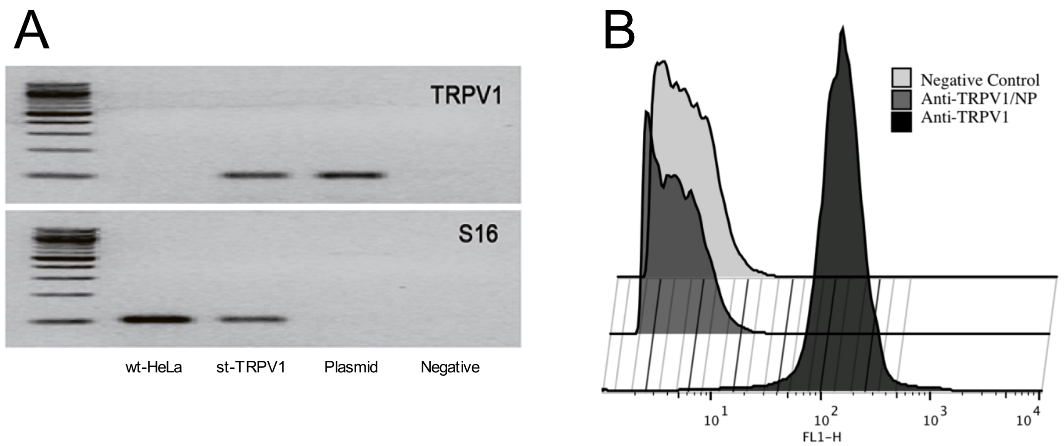

Supplementary Figure S1. TRPV1 expression in wt-HeLa and st-TRV1 HeLa cells. (A) Representative RT-PCR experiment showing TRPV1 mRNA expression (upper blot) and housekeeping S16 (lower blot) in wt-HeLa cells (lane 1), st-TRV1 HeLa cells (lane 2), plasmid containing TRPV1 gene (lane 3) and negative control in the absence of polymerase. Upper blot shows. (n=4). (B) Representative flow cytometry histogram showing st-TRV1 HeLa cells in the presence of Anti-TRPV1 in permeabilized (Anti-TRPV1) conditions (black), st-TRV1 HeLa cells in the presence of Anti-TRPV1 in non-permeabilized (Anti-TRPV1/NP) conditions (dark grey), and st-TRV1 HeLa cells in the absence of Anti-TRPV1 in permeabilized (Anti-TRPV1) conditions (grey). (n=4).

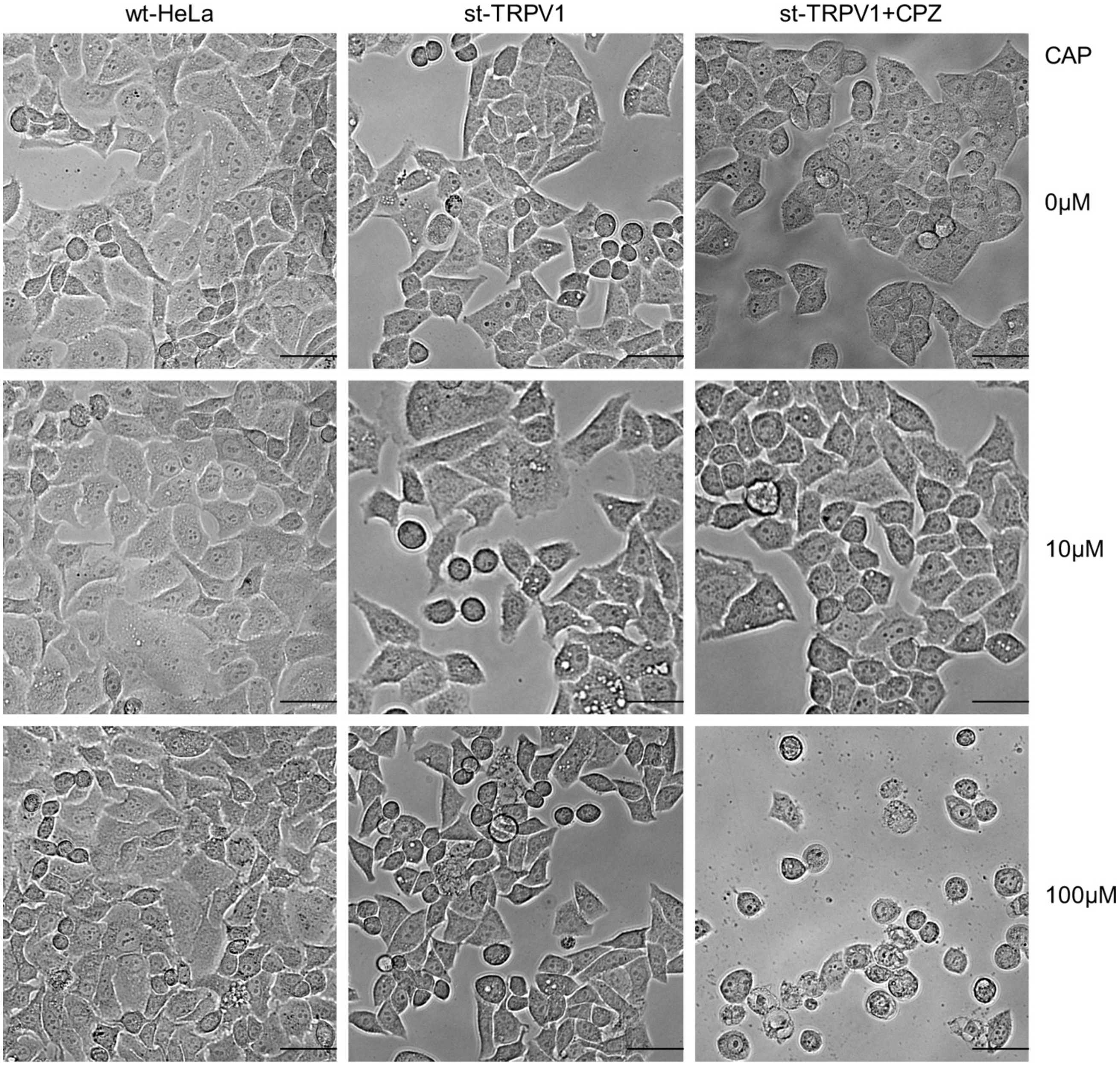

Supplementary Figure S2. TRPV1 expression increases CAP-induced cell death. Representative captures depicting wt-HeLa cells and st-TRPV1 HeLa cells exposed to 0, 10, and 100 μM CAP for 24 h in the presence or absence 10 μM CPZ. Normal cobblestone-like morphology (upper and middle-left panels) is lost after exposure to CAP in a dose-dependent manner, giving rise to a phenotype characterized by the presence of cell debris in cell media, signs of cell detachment, rounding of the cellular border, presence of vacuoles in the cytoplasm, plasma membrane bleb formation, and pyknotic nuclear fragmentation. Scale bar = 50μm.

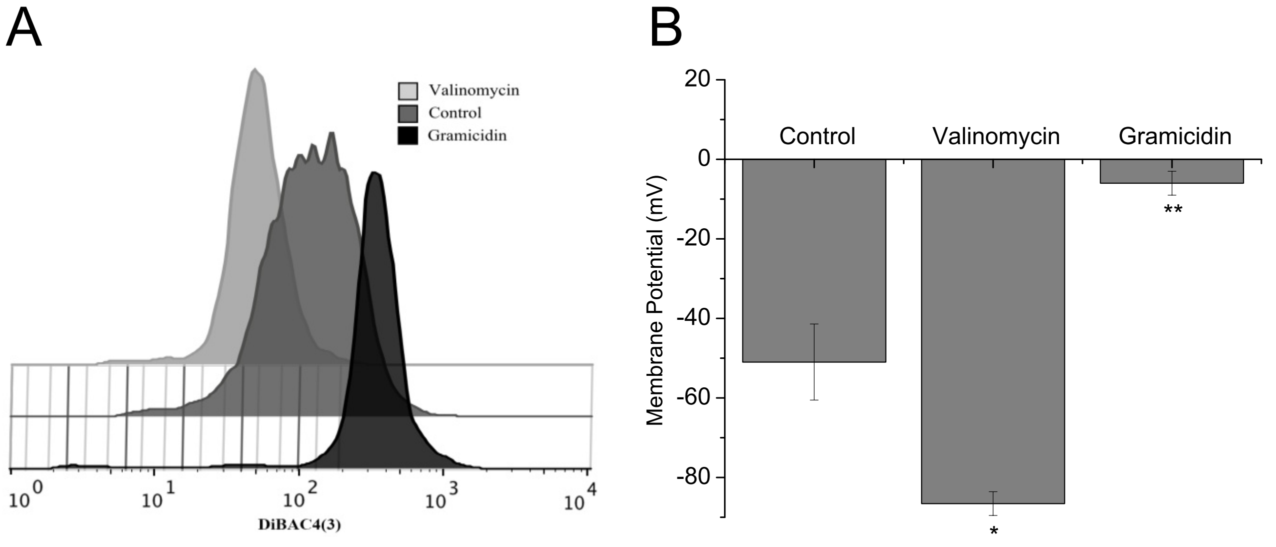

Supplementary Figure S3. DIBAC$_4$(3) efficiency to measure plasma membrane potential. (A) Representative flow cytometry histogram showing wt-HeLa cells incubated in the absence (dark grey) or in the presence of valinomycin (gray) or gramicidin (black) and then exposed to DIBAC$_4$(3). (B) Several experiments, as showed in (A), were quantified as the mean maximal fluorescence and then converted to mV (membrane potential). Data are showed as mean ± SEM. (n=5). *: $p<0.01$, **: $p<0.05$.
