# Supplementary figures and images for: Transient Receptor Potential Vanilloid 1 Expression Mediates Capsaicin-Induced Cell Death
Source: Front Physiol. 2018 Jun 5;9:682. doi: 10.3389/fphys.2018.00682 (PMC5996173; doi:10.3389/fphys.2018.00682)

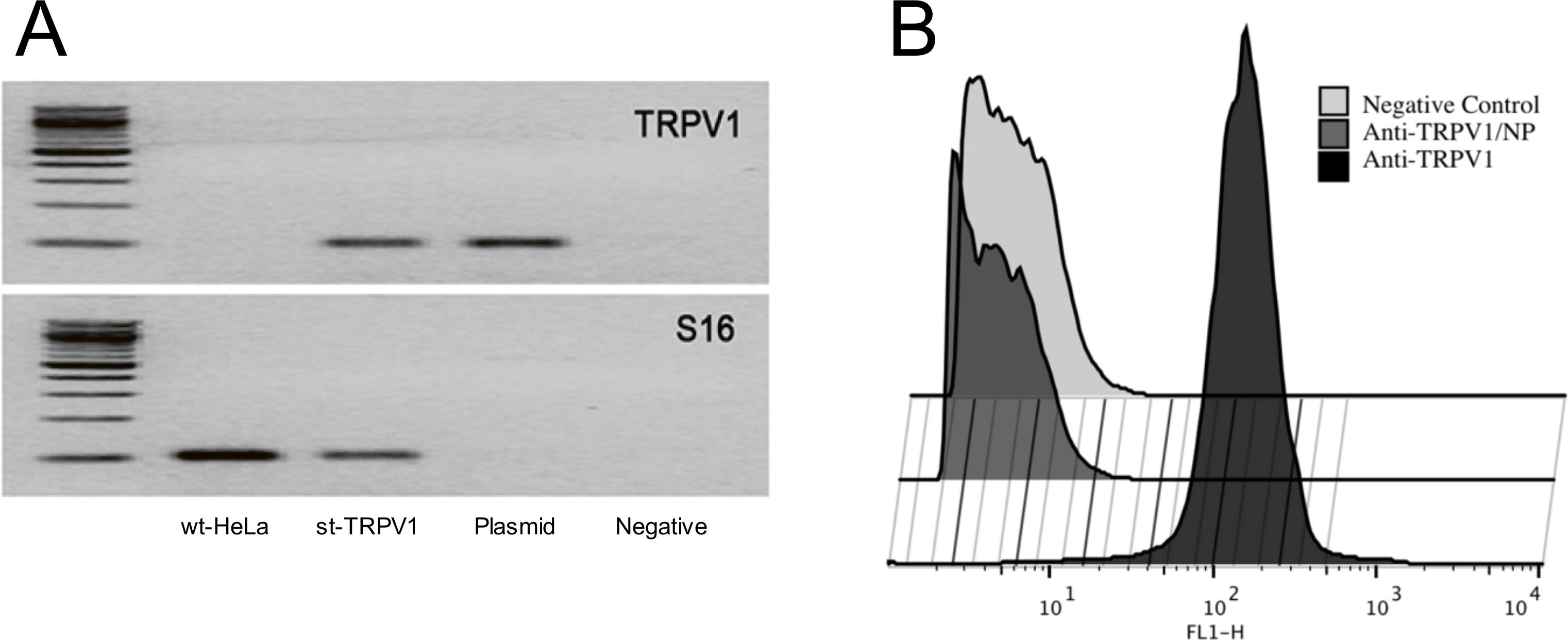

Supplement: Supplementary file 2 [file Image_1.TIF]

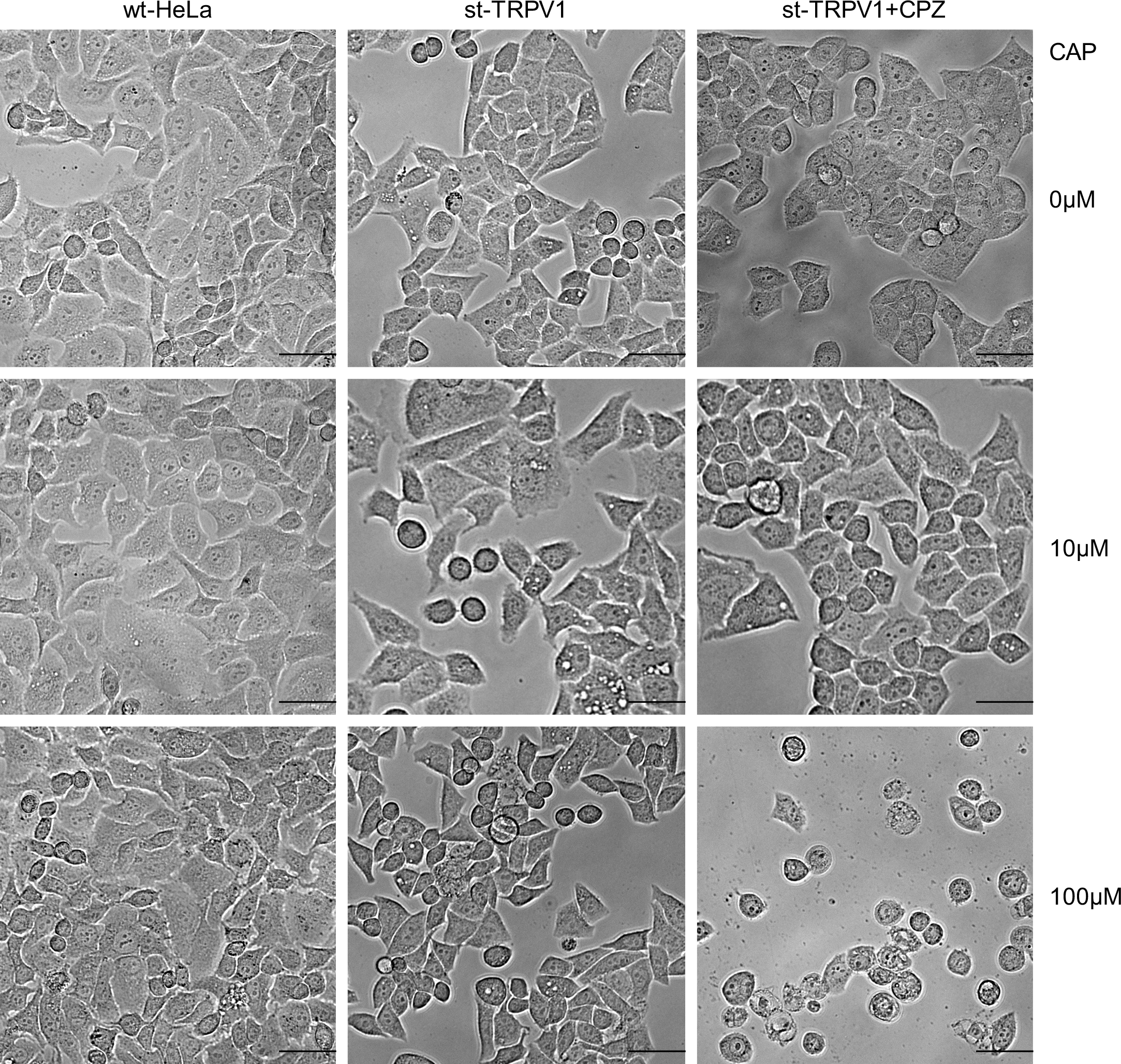

Supplement: Supplementary file 3 [file Image_2.TIF]

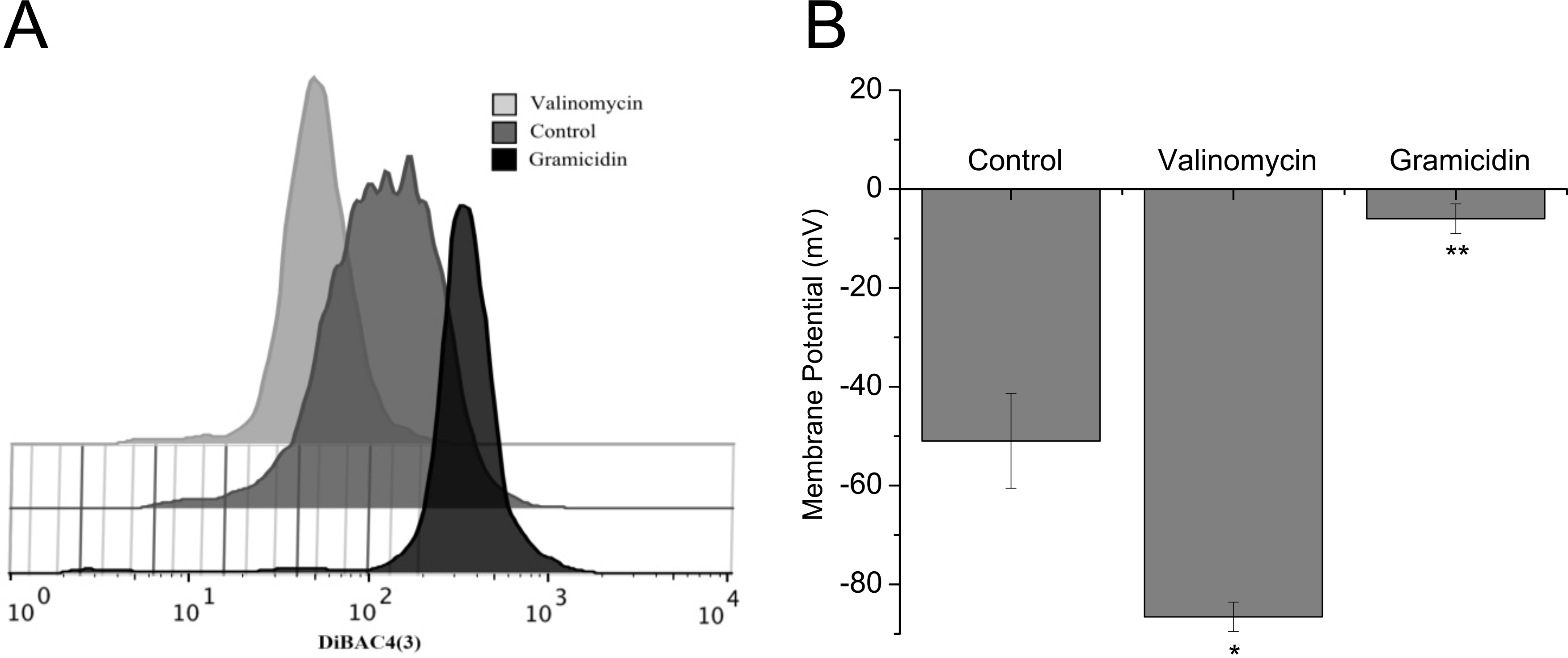

Supplement: Supplementary file 4 [file Image_3.TIF]
